# Increased Organic Fertilizer and Reduced Chemical Fertilizer Increased Fungal Diversity and the Abundance of Beneficial Fungi on the Grape Berry Surface in Arid Areas

**DOI:** 10.3389/fmicb.2021.628503

**Published:** 2021-05-07

**Authors:** Linnan Wu, Zhiqiang Li, Fengyun Zhao, Benzhou Zhao, Fesobi Olumide Phillip, Jianrong Feng, Huaifeng Liu, Kun Yu

**Affiliations:** ^1^Department of Horticulture, College of Agriculture, The Key Laboratory of Characteristics of Fruit and Vegetable Cultivation and Utilization of Germoplasm Resources of the Xinjiang Production and Construction Crops, Shihezi University, Shihezi, China; ^2^Shihezi Academy of Agricultural Sciences, Shihezi, China

**Keywords:** organic fertilizer, chemical fertilizer, fungal diversity, beneficial fungi, grape

## Abstract

Fertilizer practices can significantly impact the fruit quality and microbial diversity of the orchards. The fungi on the surface of fruits are essential for fruit storability and safety. However, it is not clear whether fertilization affects the fungal diversity and community structure on the surface of grape berries. Here, grape quality and the fungal diversity on the surface of grapes harvested from three fertilizer treatments were analyzed shortly after grape picking (T0) and following 8 days of storage (T1). The study involved three treatments: (1) common chemical fertilizer for 2 years (CH); (2) increased organic fertilizer and reduced chemical fertilizer for 1 year (A.O); and (3) increased organic fertilizer and reduced chemical fertilizer for 2 years (B.O). The application of increased organic fertilizer and reduced chemical fertilizer increased the soluble solids content (SSC) of the grape berries and decreased the pH of the grape juice. A total of 827,947 high-quality fungal sequences were recovered and assigned to 527 operational taxonomic units. Members of the *Ascomycota* phylum were dominant in all samples and accounted for 94.41% of the total number of detected sequences, followed by the *Basidiomycota* (5.05%), and unidentified fungi (0.54%). Alpha and beta diversity analyses revealed significantly different fungal populations in the three fertilizer treatments over the two time periods. The fungal diversity and richness on the grape berry surface in the B.O and A.O treatments were higher than those in the CH treatment. Among the detected fungi, the B.O treatments were mainly *Pichia*, *Aureobasidium*, and *Candida* genera, while the CH treatments were *Botrytis*, *Aspergillus*, and *Penicillium*. Moreover, significant differences were revealed between the two assessment times (T0 and T1). The samples from the T0 timepoint had higher fungal richness and diversity than the samples from T1 timepoint. Increasing organic fertilizer usage in grape management could improve grape quality and went on to increase the fungal diversity, as well as the relative abundance (RA) of beneficial fungi on grape berry surfaces. The correlation analysis suggested that the pH of the grape juice was significantly negatively correlated with fungal diversity parameters.

## Introduction

Increases in global grain and fruit yields in recent decades have been largely dependent on heavy investments in fertilizer (Geng et al., 2019). However, fertilizer can harm marine, freshwater, and terrestrial ecosystems (Tilman et al., 2011). Soil conditions with excess nutrients may result in potential damages to the environment, causing widespread environmental problems (García-Díaz et al., 2017). Excessive fertilizer application aggravated the decline in soil organic matter (SOM) and fertility (Lv et al., 2020; Wu et al., 2020). Therefore, in addition to yields, agricultural production needs to take environmental sustainability into account. Organic fertilizers are often used as basal fertilizer to ensure that SOM and micronutrients play unique roles during grapevine growth and development (Zhao et al., 2019). Organic fertilizer can affect soil moisture-holding capacity by increasing soil infiltration and minimizing soil evaporation (Moreno-Jiménez et al., 2019). Although organic fertilizers are more eco-friendly, they have low nutrient concentrations and nutrient release is too slow to support grape production in a short time (Xiao et al., 2017). The nutrient contents of chemical fertilizers are higher than that of organic ones, but they easily to cause environmental pollution and soil degradation (Song et al., 2020). The partial replacement of chemical fertilizers with organic fertilizers has proven to be a beneficial approach to sustain soil fertility compared to applying chemical or organic fertilizers alone (Song et al., 2020). Studies have shown that the use of inorganic-organic compound fertilizers can not only reduce the use of chemical fertilizer but also improve the efficiency and sustainability of agricultural ecosystems in the long term (Geng et al., 2019).

Numerous studies have found that the combination of chemical fertilizer and organic fertilizer can improve the quality of peaches (Sharma et al., 2017), pears (Wei et al., 2012), grapes (Zhao et al., 2020), and others. Organic fertilizer application increased the soluble sugar content of bananas (Zhang et al., 2020), decreased the titratable acid content of pears (Wei et al., 2012), and significantly affected the pH of the grape juice (Zhao et al., 2020). Through the modification of soil physicochemical properties, fertilization has been found to influence the soil microbial biomass and community composition (Liu et al., 2010). Similar studies also found that the use of organic fertilizer not only affected soil physical properties but also enhanced soil microbial diversity (Hao et al., 2008). Many studies have shown that organic fertilization had positive effects on soil microbial richness and diversity (Zhang et al., 2012; Geng et al., 2019). By contrast, chemical fertilization was bad for microbial diversity (Luan et al., 2020). Plant surfaces host diverse and abundant microbial communities, which are related to specific functions that may affect plant productivity and health (Angeli et al., 2019). Microbial communities associated with grape leaves, flowers, and berries shared a greater proportion of taxa with the soil communities, suggesting that soil may serve as a microbial reservoir (Zarraonaindia et al., 2015). *Ascomycota*, *Basidiomycota*, *Chytridiomycota*, *Blastocladiomycota*, and *Glomeromycota* were main fungal phylum in the soil; *Ascomycota* and *Basidiomycota* were main fungal phylum on the grape berry surface. There were 24.63% of the fungal genera which were common to soil and on the grapes surface (Morrison-Whittle et al., 2017). Martins et al. (2013) also found about 50% of the bacteria genera that were common to soil and grapes. Microbial community structure on the grape surface is mainly controlled by the cultivation environments (Abdelfattah et al., 2016a; Kecskemeti et al., 2016). The microbiota on the surface of grapes are important for the development of plant diseases and might also impact fruit maturity (Buchholz et al., 2018). Barata et al. (2012) identified over 50 bacterial species on grape berries, mainly belonging to two groups, *Proteobacteria* and *Firmicutes*. The bacterial community on the surface of grape berry changed with the ripening of the grapes (Renouf et al., 2005). The majority of research has been focused on the bacterial communities, but fungi are also important (Escribano-Viana et al., 2018). Fungi can profoundly influence plant function, health, and development, through a wide range of interactions acting as biocontrol agents or as pathogens (Oliveira et al., 2018). Some fungi are vital for plant health as well as fruit quality and yield (Ding et al., 2019). Previous studies have mostly focused on the pathogenic fungi causing grape diseases, including *Botrytis cinerea* (Chen et al., 2019), *Penicillium expansum* (Jurick 2nd et al., 2020; Luciano-Rosario et al., 2020), and *Aspergillus japonicus* (Yan and Liu, 2019), the causal agents of grapevine powdery mildew, blue mold, and bitter rot, respectively (Lorenzini et al., 2018). Moreover, researchers have also identified saprophytic molds, such as *Cladosporium* sp., *Aspergillus* sp., and *Penicillium* sp., that could produce mycotoxins, which were indirectly responsible for food spoilage and directly responsible for grape rot (Martins et al., 2014). Several *Botryosphaeriaceae* species were known to occur worldwide, causing dieback, canker, and fruit rot on various hosts (Akila et al., 2020). Grape black rot is caused by *Guignardia bidwellii* and was the grapevine disease attributed to a *botryosphaeriaceous* fungus (Onesti et al., 2017). Macrophoma rot is caused by *Botryosphaeria dothidea* (Úrbez-Torres, 2011; Xu et al., 2015). Both *Botryosphaeria kuwatsukai* and *B. dothidea* are the main causal agents for apple ring rot in China and Japan (Xu et al., 2015). Plant-associated fungi, such as *Aureobasidium pullulans* (Lorenzini and Zapparoli, 2019) and *Candida xylopsoci* (Gao et al., 2019), have also been suggested to have a positive interaction with their host plants. In the production of organic apples and pears, *A. pullulans* is used as a biocontrol agent for fire blight protection (Temple et al., 2020). Utilization of *A. pullulans* was shown to be successful in controlling against both *P. expansum* and *Penicillium digitatum* (Agirman and Erten, 2020). A better understanding of these interactions may provide novel opportunities to develop creative biocontrol methods against plant pathogens (Sebastien et al., 2015). Contemporary studies dealing with the microbiome in grapes and the winemaking process have focused on the effects of vineyard locations and grape varieties (Shen et al., 2020). Subsequently, varieties, management methods, and eco-geographic factors were taken into account to explain their possible impacts on the microbiome associated with grapes (Bokulich et al., 2014). Fungi associated with the winemaking process and grape musts have been extensively documented (Escribano-Viana et al., 2018; Oliveira et al., 2018), however, few studies have concentrated on the fungal community composition on the table grape berry surface under different fertilizer practices.

Xinjiang is an area with a long history of viticulture, with a planting area of 142,900 ha (Huang, 2019). Located in arid regions in Xinjiang, the orchard environment is fragile, and the long-term unscientific use of chemical fertilizer results in an increased frequency of fungal diseases such as powdery mildew (*Erysiphe necator*) and downy mildew (*Plasmopara viticola*) in vineyards. Previous studies mainly focused on the effects of fertilization on the growth of grapes (Liu et al., 2016) and the soil environment (Zhang et al., 2018; Cheng et al., 2020). The fungi on the surface of the grape berries are essential for the quality and storage of grape berries, which are also very important for people’s food safety. However, there is no report on the effects of different fertilization modes on grape berry surface fungal communities. The paper aimed to investigate the grape quality and the fungal diversity on the grape berry surface under three fertilizer treatments at two postharvest timepoints to provide a theoretical basis for improving the ecological environment and promoting the sustainable production of grapes in arid areas.

## Materials and Methods

### Experimental Site and Experimental Design

The experimental site was located in the grape standard experimental orchard (lat. 45°19'N, long. 86°03'E) of Shihezi Agricultural Science Research Institute in Xinjiang. The experimental zone is classified as temperate and arid, with a continental climate. The site’s annual precipitation, annual evaporation, and mean annual temperature are 0.23 m, 1.34 m, and 7.8°C, respectively (Tao et al., 2018). The meteorological data (daily temperature and daily rainfall) recorded during the experiment are shown in Supplementary Figure S1. The soil was sandy loam soil. The basic soil properties before the beginning of the experiment were as follow: pH, 8.04; SOM, 32.23 g kg^−1^; total nitrogen (TN), 1.26 g kg^−1^; alkali-hydrolysed nitrogen (AN), 50.13 mg kg^−1^; total phosphorus (TP), 0.89 g kg^−1^; available phosphorus (AP), 42.95 mg kg^−1^; available potassium (AK), 121.05 mg kg^−1^; and conductivity, 2.10 ms m^−1^. The soil pH, SOM, TN, TP, TK, AN, AK, and AP was measured as described (Bao, 2000).

**Figure 1:**
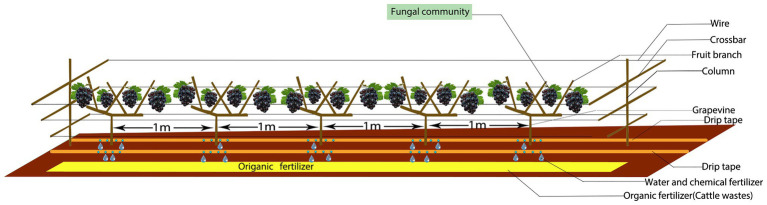
Experimental model.

These experiments used table grapes (*Vitis vinifera* L.) from one cultivar (Summer Black) that is widely planted in Xinjiang. Each treatment was repeated three times and randomly assigned to the experimental units. The grape trellis was a V-shaped scaffold (Figure 1) placed between the vines. In the spring, organic fertilizer was applied as the base fertilizer, 0.25 m deep (unilateral fertilization, and on one side of the grape line to avoid mutual influence between the grape plants) and 0.30 m away from the central trunk of the grape vine. Chemical fertilizers (drip application) were applied during the growing season. A total of 11 irrigations were carried out during the whole growth period, and the irrigation interval was 7–10 days. The three fertilization treatments had the same irrigation dates and irrigation times. The three treatments were: (1) common chemical fertilizer for 2 years (CH); (2) increased organic fertilizer and reduced chemical fertilizer for 1 year (A.O); and (3) increased organic fertilizer and reduce chemical fertilizer for 2 years (B.O). The study used a randomized complete block designed with three replicates of the three treatments. Rotten cattle manure was used as organic fertilizer containing 2.48% nitrogen, 1.79% phosphorus, and 1.67% potassium. See Table 1 for the application amount. The amount of chemical fertilizer applied in the CH treatment was determined according to the amount of fertilizer used by local fruit farmers on the vineyard.

**Table 1:** Specific fertilizer application amounts for different treatments and years.

	2017				2018			
Treatment	Chemical fertilizer (kg hm^−2^)			Base fertilizer (kg hm^−2^)	Chemical fertilizer (kg hm^−2^)			Base fertilizer (kg hm^−2^)
	N	P_2_O_5_	K_2_O	Organic fertilizer	N	P_2_O_5_	K_2_O	Organic fertilizer
CH	289.8	172.5	345	0	289.8	172.5	345	0
A.O	217.4	129.3	258.8	0	217.4	129.3	258.8	2921.4
B.O	217.4	129.3	258.8	2921.4	217.4	129.3	258.8	0

The organic fertilizer application amount is consistent with the pure nitrogen nutrient amount provided by chemical fertilizer.

### Sample Preparation

Grape bunches were chosen to represent the same aspect (orientation to prevailing wind and sun) and position (proximity to the middle of the row), and samples were randomly collected from each vine at 11 a.m. on August 20, 2018. Then, samples were transported to the laboratory, and healthy bunches were selected based on color uniformity and absence of blemishes or disease. The first nine samples (three replicates × three treatments) were analyzed for grape quality and fungal communities on the grape berry surface shortly after grape picking. The other samples of the three fertilizer treatments (five clusters per fertilizer treatment) were packed into three cardboard boxes and stored at room temperature (~20°C) for 8 days. Table grapes can be stored for 7–12 days at room temperature and some table grapes are sold during this period (Zhu, 2020). Eighteen samples (three replicates × three treatments × two timepoints) of microbial DNA were collected from the grape surfaces. Each treatment consisted of three replications with 50 fresh grape berries per replicate. The microbial samples were gathered by wiping or swabbing each grape with a pre-moistened cotton swab. Swab samples were collected and stored at −80°C before microbial DNA extraction. The wiped grape berries were then used to assess the quality parameters of the grape berries.

### Determination of Quality Parameters of Grape Berries

The pH was determined using a Mettler Toledo FE20 Desktop pH Meter (Mettler Toledo Instruments Co. Ltd., Shanghai, China). The SSC was measured with a Pocket Brix-acidity Meter (Atago, PALBX/ACID 5, Tokyo, Japan). Titratable acid content was determined using potentiometric titration with 0.1 N NaOH up to pH = 8.2 and expressed as percentage of tartaric acid (Youssef et al., 2015). The total anthocyanin content (TAC) in berries was determined by the pH differential method (Yang et al., 2020). The berry firmness was performed with a texture analyzer (TA). XT plus (Stable Micro Systems, Surrey, England), analyzing the equatorial position of 15 berries with pedicels per plot (Youssef et al., 2015).

### Extraction of Genomeice DNA and PCR Amplification

The MoBio Power Water® DNA Isolation kit (MoBio Laboratories, Inc., Carlsbad, CA, United States) was used to extract microbial genomic DNA from the swabs. The NC 2000 spectrophotometer (Thermo Scientific Fisher, Waltham, MA, United States) and 1.0% agarose gel electrophoresis were used to determine the concentration and molecular size of the extracted DNA, respectively. Then, the DNA extractions were stored at −80°C until further use. Grape berries were analyzed for fungal communities shortly after grape picking, which was referred to as T0. Grape samples stored for 8 days, were referred to as T1. A total of 18 diluted DNA samples were submitted to Shanghai Personal Biotechnology Co., Ltd (Shanghai, China) for internal transcribed spacer (ITS) sequencing using the Illumina HiSeq sequencing platform. The specific pairs were ITS25F (5'-GGAAGTAAAAGTAACAAGG-3') and ITS1R (5'-GCTGCGTTCTTCATGC-3'). The PCR reaction contained 5 U of DNA polymerase (Pyrobest TaKaRa, Japan), 15 pmol of each primer, 2.5 mM of dNTP mixture, 10 μl of 10x Buffer II, and 40 ng of template DNA for a total volume of 25 μl. The PCR amplification series was performed using an ABI 9600 instrument under method of Hao et al. (2016). The obtained ITS1 amplicons were sequenced on the Illumina MiSeq platform at Shanghai Personal Biotechnology Co., Ltd (Shanghai, China).

### PCR Product Purification and Library Preparation

According to the concentrations of the PCR products, PCR products were mixed at equal density ratios and then purified using a Qiagen Gel Extraction Kit (Qiagen Company in Germany; Zetsche et al., 2017). The TruSeq® DNA PCR-Free Sample Preparation Kit (Illumina, United States) was used to generate sequencing libraries, and then index codes were added (Noguerol-Pato et al., 2014). The library quality was evaluated using the Qubit@ 2.0 Fluorometer (Thermo Scientific) and an Agilent Bioanalyzer 2100 system (Kao et al., 2016). Finally, the library was sequenced on the Illumina HiSeq 2500 platform and 250 bp paired-end reads were generated.

### Bioinformatic Analyses

After completing high-throughput sequencing, paired-end reads were obtained, which were assigned to samples based on their unique barcodes, and then truncated by cutting off the barcode and primer sequences. Using FLASH V1.2.7 spliced the paired-end reads (El-Ashram and Suo, 2017), filtered according to the QIIME V1.7.0 quality control process, and termed the high-quality clean tags. The tags were compared to the reference database using the UCHIME algorithm to detect chimeric sequences, and then removed the chimeric sequences (Li et al., 2018). Finally, we obtained the effective tags. Alpha diversity was evaluated using the QIIME suite of programmes, including the Simpson index, ACE, Shannon diversity, Chao1 richness, and observed species (Marsh et al., 2013). Principal coordinate analysis (PCoA) based on weighted calculations was used to analyze the differences in the community structure of the different treatments and timepoints (Horn et al., 2017). We used flower charts and Venn diagrams to explore the specific and common operational taxonomic units (OTUs) among the different treatments or timepoints. The microbial communities on the grape berry surfaces from the different treatments/timepoints were further compared using analysis of similarities (Anosim) and analysis of molecular variance (Amova). Amova was used to assess the significance of the fungal community structures among the different treatments. After basic analysis, Origin 2019 and R Studio (version 2.15.3) were used to draw figures (Hayden and Beman, 2016).

### Statistical Analysis

SPSS 20.0 software was used to test the significance of the differences in the quality and fungal diversity of grapes, and the data were expressed as the means ± SD of triplicates.

## Result

### The Quality Parameters of Grape Berries

**Figure 2:**
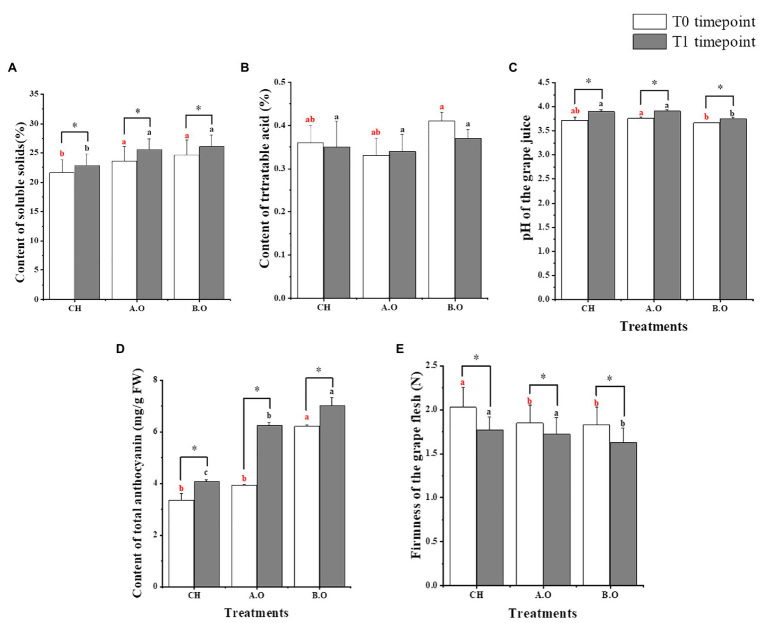
Quality parameters of grape berries. **(A)** Soluble solids content (SSC) of grape berries. **(B)** Titratable acid contents. **(C)** The pH of the grape juice. **(D)** Total anthocyanins contents. **(E)** Firmness of the grape flesh. Values presented are the means ± SD (*n* ≥ 3). Vertical bars indicate the SD of the means. Different letters indicate significant differences among treatments from the same timepoint using Duncan’s test (*p* < 0.05). ^∗^ indicates a significant difference between the T0 and T1 timepoint samples of the same treatment using a one-tailed Student’s *t*-test (*p* < 0.05).

The SSC, pH of the grape juice, and TAC significantly increased after 8 days of storage in grapes from all three fertilizer treatments. At both timepoints, the SSC was significantly higher with the organic fertilizer treatments (A.O and B.O) relative to the chemical fertilizer treatment (CH); the value of the B.O sample was 14.21% higher than that of the CH sample, and the value of the A.O sample increased by 11.76% compared with the CH samples from T1 timepoint (Figure 2A). Of the three treatments, the titratable acid content was the highest in grapes from the B.O treatment at both the T0 and T1 timepoints (Figure 2B). Figure 2C shows the effect of the fertilizer treatments on the pH of the grape juice. At both the T0 and T1 timepoints, the pH of the grape juice in the A.O samples was significantly higher than that in the B.O samples. As seen in Figure 2D, at both timepoints, the TAC of the B.O and A.O samples was higher than that of the CH sample. The TAC in the T1.B.O and T1.A.O samples increased by 72.48 and 53.32%, respectively, compared with the T1.CH samples. Figure 2E shows that the firmness of the grape flesh from the three fertilizer treatments decreased during the storage period. At both timepoints, the firmness of grape flesh in the CH samples was the highest, while the B.O samples were the lowest. The firmness of the grape flesh in the CH samples increased by 8.87 and 9.85% compared with A.O and B.O samples, respectively, at the T0 timepoint.

### ITS-Based Fungal Sequencing Data and Fungal Diversity

A total of 848,903 fungal ITS sequences passed quality control. The total number of reads obtained was 415,995 in the grape samples obtained at T0 timepoint and 432,909 in the samples at the T1 timepoint. Then, the high-quality sequences were clustered into OTUs at 97% sequence identity. After filtering rare OTUs, a total of 827,947 sequences were clustered into 527 identified OTUs (Supplementary Table S1).

The observed species and Shannon and ACE indexes of the three fertilizer treatment samples were significantly higher at the T0 timepoint than those at the T1 timepoint. At the T0 timepoint, the Shannon index of the B.O and A.O samples was significantly increased by 14.38 and 11.71%, respectively, compared to the CH samples. The observed species and ACE index of the B.O samples were the highest of three fertilizer treatments. At the T1 timepoint, the Shannon index of the B.O samples was higher than that of the A.O and CH samples, and the difference between the B.O samples and the CH samples was significant. The observed species and ACE index were significantly higher in the B.O samples relative to the A.O and CH samples (Table 2).

**Table 2:** Richness and diversity indexes of the fungal communities on the grape berry surfaces following the three treatments across the two timepoints.

Timepoint	Treatment	Reads	Observed species	Diversity and richness indexes			
				Shannon	Simpson	Chao1	ACE
T0	CH	45,316 ± 3246a	208 ± 15b^∗^	2.99 ± 0.06b^∗^	0.82 ± 0.02a^∗^	228.67 ± 8.08a^∗^	216.67 ± 6.66b^∗^
	A.O	46,222 ± 2490a	227 ± 7ab^∗^	3.34 ± 0.14a^∗^	0.83 ± 0.02a	227.50 ± 7.96a	229.44 ± 9.41ab^∗^
	B.O	47,127 ± 3736a	236 ± 7a^∗^	3.42 ± 0.14a^∗^	0.81 ± 0.02a	232.68 ± 8.08a	233.45 ± 7.77a^∗^
T1	CH	47,199 ± 5444a	145 ± 35b^∗^	2.33 ± 0.45b^∗^	0.66 ± 0.03b^∗^	149.71 ± 23.91b^∗^	153.71 ± 25.07b^∗^
	A.O	47,698 ± 2024a	169 ± 17b^∗^	2.55 ± 0.19ab^∗^	0.74 ± 0.01a	160.31 ± 5.53b	161.63 ± 3.82b^∗^
	B.O	49,405 ± 6490a	218 ± 13a^∗^	2.95 ± 0.12a^∗^	0.75 ± 0.02a	193.00 ± 9.85a	212.92 ± 10.68a^∗^

Different lowercase letters after data within the same column and timepoint indicate significant differences between the treatments (*p* < 0.05; Duncan’s test). All of the values are expressed as the means ± SD of three replicates.

∗ Significant differences between the T0 and T1 timepoints of the same treatment, as determined by a one-tailed Student’s *t*-test (*p* < 0.05).

### Overall Characteristics of the Fungal Community

**Figure 3:**
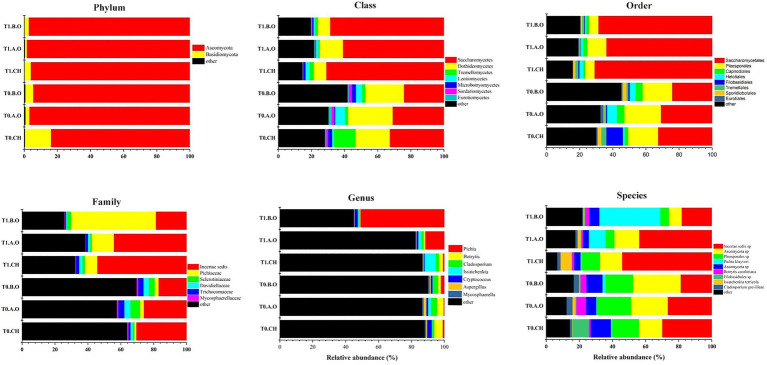
Composition of predominant fungi (abundance ≥ 0.5%) at different taxonomic levels (phylum, class, order, family, genus, and species) from the three fertilizer treatments at the T0 and T1 timepoints.

The percentages and distributions of the predominant fungi at different classification levels (phylum, class, order, family, genus, and species) are shown in Figure 3. *Ascomycota* was the most abundant phylum, accounting for 94.41% of the total sequences. We also detected *Basidiomycota* as a minor phylum (5.05%). Dominant classes included *Saccharomycetes* (48.47%), *Dothideomycetes* (16.63%), and *Tremellomycetes* (3.65%). Dominant orders were *Saccharomycetales* (48.47%), *Pleosporales* (13.46%), *Capnodiales* (2.63%), and *Helotials* (2.57%). Dominant families were *Pichiaceae* (13.01%), *Sclerotiniaceae* (2.57%), and *Davidiellaceae* (2.03%). A total of 99 genera were detected in grape surface samples from the T0 timepoint, and 83 genera were detected in the grape surface samples from the T1 timepoint. The predominant genera of samples from the T0 timepoint were *Botrytis* (3.55%), *Cladosporium* (2.72%), *Cryptococcus* (1.68%), *Pichia* (1.06%), *Aspergillus* (0.98%), and *Aureobasidium* (0.73%). Correspondingly, the predominant genera of samples from the T1 timepoint were *Pichia* (21.26%), *Issatchenkia* (3.07%), *Botryis* (1.58%), *Cladosporium* (1.34%), *Cryptococcus* (1.17%), *Aureobasidium* (0.39%), and *Aspergillus* (0.32%; Table 3). Most of these highly detected genera included multiple species. A total of nine species *Cryptococcus* were detected, primarily *C. albidus*, *C. cyanovorans*, *C. heimaeyensis*, and *C. heveanensis*. *Aspergillus* was detected with nine species, mainly of *A. japonicus*, *A. pipers*, *A. cibarius*, *A. subversicolor*, and *A. ochraceus*. Two species of *Aureobasidium*, *A. pullulans* and *A. microstictum* were highly detected in all samples. A total of seven species of *Penicillium* were detected primarily *P. spinulosum*, *P. cinnamopurpureum*, and *P. bialowiezense*. Only one species each of *Pichia*, *Issatchenkia*, *Botrytis*, and *Cladosporium* were detected, *P. kluyveri*, *I. terricola*, *B. caroliniana*, and *C. grevilleae*, respectively (Supplementary Table S1).

**Table 3:** Effects of storage time and fertilizer treatment on the fungal communities on the grape berry surface at the genus level.

Genus (%)	T0			T1		
	CH	A.O	B.O	CH	A.O	B.O
*Pichia*	0.87 ± 0.21ab	0.33 ± 0.06b^∗^	1.97 ± 0.23a^∗^	0.77 ± 0.40c	11.50 ± 5.64b^∗^	51.80 ± 15.01a^∗^
*Botrytis*	5.97 ± 2.22a^∗^	3.63 ± 1.84ab^∗^	1.06 ± 0.25b	2.43 ± 0.78a^∗^	1.40 ± 0.53ab^∗^	0.90 ± 0.20b
*Cladosporium*	3.63 ± 0.45a^∗^	3.23 ± 1.26a^∗^	1.30 ± 0.30b	2.03 ± 0.46a^∗^	1.37 ± 0.40ab^∗^	0.63 ± 0.35b
*Cryptococcus*	2.87 ± 1.00a	1.07 ± 0.23b	1.10 ± 0.01b	1.87 ± 1.80a	1.07 ± 0.11b	0.57 ± 0.31b
*Aspergillus*	1.63 ± 0.19a^∗^	0.67 ± 0.40b	0.63 ± 0.21b	0.37 ± 0.21a^∗^	0.37 ± 0.11ab	0.23 ± 0.05b
*Aureobasidium*	0.43 ± 0.17b	0.63 ± 0.27ab^∗^	1.13 ± 71a	0.12 ± 0.01b	0.17 ± 0.06b^∗^	0.87 ± 0.25a^∗^
*Penicillium*	0.16 ± 0.07a^∗^	0.09 ± 0.04ab	0.06 ± 0.01b	0.08 ± 0.03a^∗^	0.04 ± 0.03b	0.03 ± 0.02b
*Candida*	0.08 ± 0.07c	0.11 ± 0.02b^∗^	0.20 ± 0.01a^∗^	0.09 ± 0.04b	0.23 ± 0.02ab^∗^	0.27 ± 0.01a^∗^

Only fungal genera contributing to at least 0.05% of the total reads with significant differences between the three treatments within one period are shown. Different lowercase letters after data within the same row and timepoint indicate significant differences between treatments (*p* < 0.05; Duncan’s test). All of the values are expressed as the means ± SD of three replicates.

∗ Significant differences between the T0 and T1 timepoint within the same treatment, as determined by a one-tailed Student’s *t*-test (*p* < 0.05).

### Fungal Community Variation

**Figure 4:**
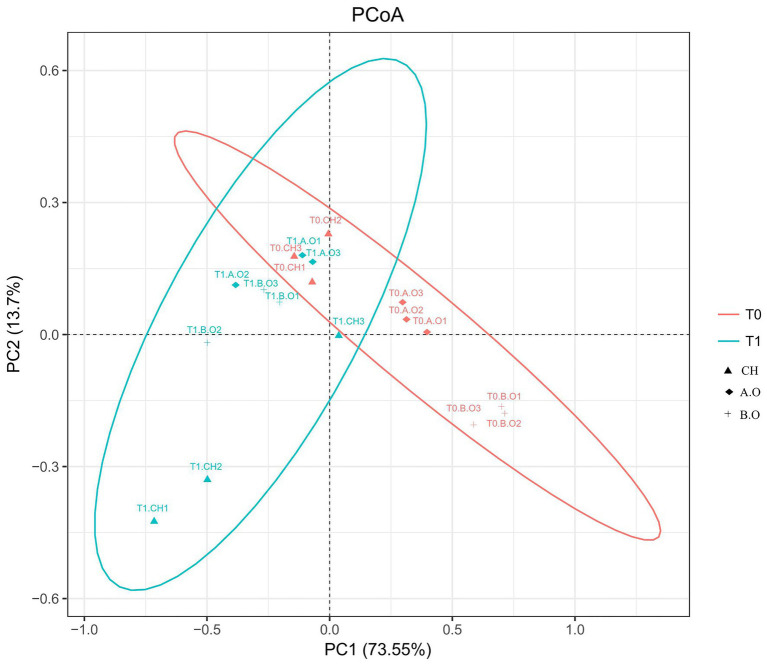
Comparison of fungal communities on the grape berry surface. A principal coordinate analysis (PCoA) based on weighted UniFrac distances was generated with operational taxonomic units (OTUs) (at 97% similarity) present in the three fertilizer treatments. Red colored oval represents samples from the T0 timepoint. Blue colored oval represents samples from the T1 timepoint.

The beta diversity results of the weighted UniFrac PCoA showed significant fungal compositional differences between samples from the T0 and T1 timepoints (Figure 4). The weighted UniFrac PCoA clustered the samples into two groups; the grape surface fungal community compositions from the T1.B.O, T1.A.O, and T0.CH samples grouped together, while the other treatments were scattered (Figure 4). PC1 contributed 73.55% and PC2 contributed 13.70%.

### Core Fungal Communities on the Surface of Grape Berries

**Figure 5:**
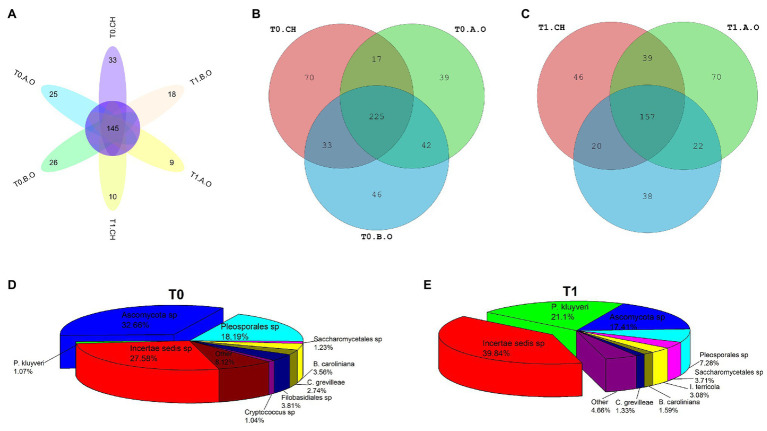
The exclusive and common fungal OTUs on the grape berry surface. **(A)** Flower chart of the three fertilizer treatments at the T0 and T1 timepoints; **(B)** Venn diagram of the three fertilizer treatments at the T0 timepoint; **(C)** Venn diagram of the three fertilizer treatments at the T1 timepoint; **(D)** Composition of the core fungal community of the three fertilizer treatments at the T0 timepoint; and **(E)** Composition of the core fungal community of the three fertilizer treatments at the T1 timepoint.

A total of 157 fungal OTUs were shared by all samples of the three treatments at the T0 and T1 timepoints (Figure 5A). There were more OTUs in the samples from the T0 timepoint (472 OTUs) than that of the samples from T1 timepoint (390 OTUs). From the total number of reads at the T0 timepoint, 345 OTUs were identified in the CH treatment, while 323 and 346 OTUs were identified in the A.O and B.O treatments (Figure 5B), respectively. A total of 225 fungal OTUs were shared by all the samples at T0 timepoint. For the T1 timepoint, 262, 288, and 237 OTUs were identified in B.O, A.O, and CH treatments (Figure 5C), respectively, and 157 fungal OTUs were shared by all the samples at the T1 timepoint. There were significantly more OTUs in the three fertilizer treatments at the T0 timepoint than at the T1 timepoint. The structure of the core fungal community was significantly different between the three treatments at both the T0 and T1 timepoints (Figures 5D,E). The dominant species of the core fungal communities of the samples at the T0 timepoint were *Ascomycota* sp., *Pleosporales* sp., *Filobasidiales* sp., and *Botrytis caroliniana*, while those of the samples at the T1 timepoint were *Pichia kluyveri*, *Ascomycota* sp., *Pleosporales* sp., and *Saccharomycetales* sp.

### The Fungal Communities at the Genus Level

At the genus level, the relative abundances (RAs) of *Pichia* and *Candida* in the organic fertilizer treatments significantly increased after 8 days of storage. By contrast, the RAs of *Botrytis*, *Cladosporium*, *Cryptococcus*, *Aspergillus*, *Aureobasidium*, and *Penicillium* in the three fertilizer treatments decreased during the storage period (Table 3). The RAs of *Botrytis* in the T0.CH and T0.A.O samples significantly decreased by 59.30 and 61.43%, respectively, compared with the T1.CH and T1.A.O samples. The RAs of *Aureobasidium* and *Candida* were the highest of three fertilizer treatments in the B.O samples at both timepoints. The RA of *Aureobasidium* in the T0.B.O samples was 162.79% higher than that in the T0.CH samples. The RAs of *Botrytis*, *Cladosporium*, and *Penicillium* in the T0.CH samples were significantly higher than those in T0.B.O samples. The RA of *Botrytis* in T0.B.O samples was 82.42% lower than that in the T0.CH samples. The RAs of *Cryptococcus* and *Aspergillus* were significantly higher in the T0.CH samples relative to the T0.B.O and T0.A.O samples. The RA of *Aspergillus* in the T0.CH samples increased by 61.35 and 58.90% compared to the T0.B.O and T0.A.O samples, respectively (Table 3).

### The Fungal Communities at the Species Level

The dominant fungi at species level were *Ascomycota* sp., *Pleosporales* sp., and *P. kluyver* at both timepoints (Figures 5D,E). However, the RAs of these fungi within the fungal community changed. Table 4 shows that the RA of *P. kluyver* in all three treatments at the T1 timepoint was higher than that at the T0 timepoint, and the same was true for *I. terricola* and *B. caroliniana*. The RA of *P. kluyver* in the T1.A.O and T1.B.O samples was significantly increased by 3509.52 and 1976.68%, respectively, compared to the T0.A.O and T0.B.O samples (Table 4). By contrast, the RAs of *Filobasidiales* sp., *A. pullulans*, *C. xylopsoci*, and *A. japonicas* in the three fertilizer treatments decreased during the storage period. *Aureobasidium pullulans* and *C. xylopsoci* RAs were the highest of three fertilizer treatments in the B.O samples at both timepoints. *Aureobasidium pullulans*, *Filpbasidiales* sp., and *C. xylopsoci* RAs were the lowest of three fertilizer treatments in the CH samples at both timepoints. The *A. pullulans* RA in the T0.B.O samples increased by 464.29% compared with the T0.CH samples. The abundance of *A. pullulans* in the T1.B.O samples was 687.50% higher than that in the T1.CH samples. The *B. caroliniana* and *A. japonicus* RAs in the T0.CH samples were significantly higher than those in the T0.B.O samples (*p* < 0.05). The *B. caroliniana* RA in the T0.B.O samples decreased by 59.73% compared with the T0.CH samples. The *I. terricola* RA was significantly higher in the T0.CH samples relative to the T0.B.O and T0.A.O samples. The *I. terricola* RA in the T0.CH samples increased by 89.15 and 85.27% compared to the T0.A.O and T0.B.O samples, respectively (Table 4).

**Table 4:** Effects of storage time and fertilizer treatment on the fungal communities on the grape berry surface at the species level.

Species (%)	T0			T1		
	CH	A.O	B.O	CH	A.O	B.O
*P. kluyver*	0.52 ± 0.17ab	0.21 ± 0.03b^∗^	1.33 ± 0.17a^∗^	0.53 ± 0.27b	7.58 ± 4.31ab^∗^	27.62 ± 18.71a^∗^
*I. terricola*	1.29 ± 0.72a^∗^	0.14 ± 0.20b^∗^	0.19 ± 0.0.07b^∗^	19.79 ± 2.73a^∗^	5.51 ± 0.71b^∗^	1.65 ± 1.10c^∗^
*B. caroliniana*	2.93 ± 0.49a^∗^	1.78 ± 0.06b^∗^	1.18 ± 0.35bc	7.34 ± 2.27a^∗^	4.37 ± 2.01ab^∗^	1.31 ± 0.22b
*Filobasidiales* sp.	0.43 ± 0.23b	0.56 ± 0.60b	7.49 ± 0.69a	0.12 ± 0.01b	0.36 ± 0.13ab	0.49 ± 0.04a
*A. pullulans*	0.14 ± 0.03c	0.30 ± 0.05b	0.79 ± 0.21a	0.08 ± 0.01b	0.08 ± 0.03b	0.63 ± 0.02a
*C. xylopsoci*	0.05 ± 0.03b	0.17 ± 0.05ab	0.20 ± 0.06a	0.09 ± 0.04b	0.08 ± 0.03b	0.12 ± 0.03a
*A. japonicus*	0.19 ± 0.012a	0.18 ± 0.02a	0.12 ± 0.03b	0.07 ± 0.02a	0.06 ± 0.00ab	0.05 ± 0.01b

Only fungal species contributing at least 0.05% of the total reads with significant differences between the three treatments within a timepoint are shown. Different lowercase letters after data within the same row and timepoint indicate significant differences between treatments (*p* < 0.05; Duncan’s test). All of the values are expressed as the means ± SD of three replicates. *P. kluyver*, *Pichia kluyver*; *I. terricola*, *Issatchenkia terricola*; *B. caroliniana*, *Botrytis caroliniana*; *A. pullulans*, *Aureobasidium pullulans*; *C. xylopsoci*, *Candida xylopsoci*; and *A. japonicas*, *Aspergillus japonicus*.

∗ Significant differences between the T0 and T1 timepoints within the same treatment, as determined by a one-tailed Student’s *t*-test (*p* < 0.05).

### Association Networks Among Fungal Species

**Figure 6:**
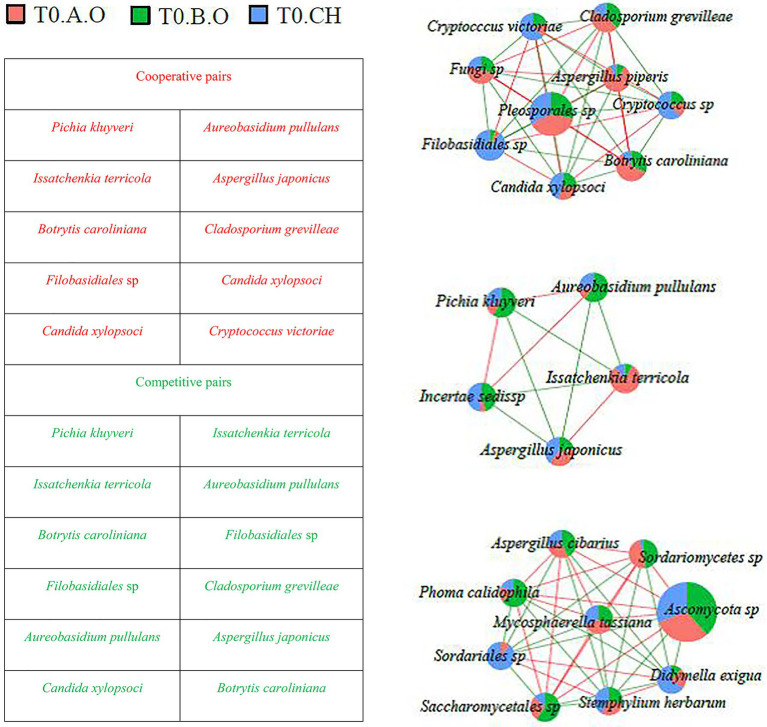
Network diagram of dominant species in the T0 timepoint samples (RA > 0.1%) showing cooperative and competitive associations. Green represents a competitive association and red represents a cooperative association.

**Figure 7:**
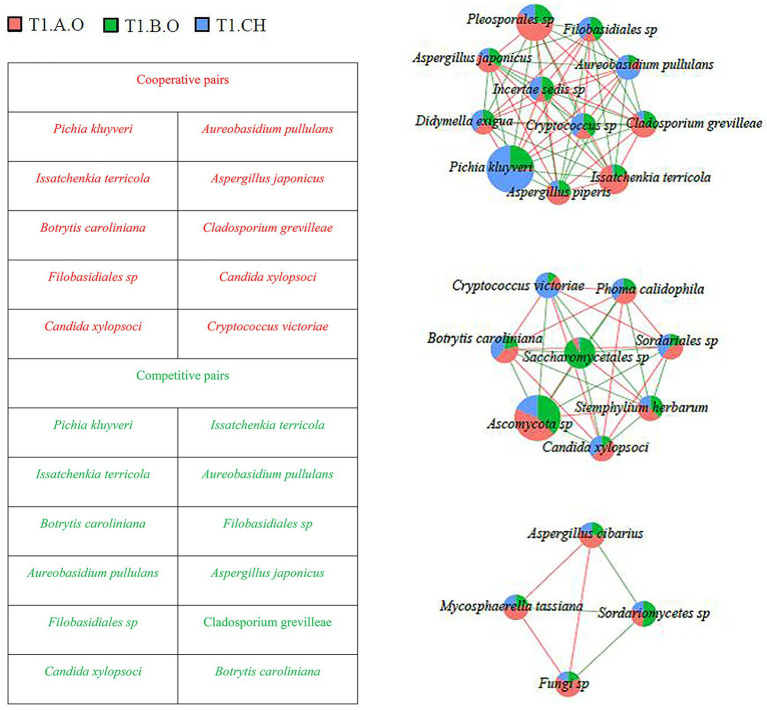
Network diagram of dominant species in the T1 timepoint samples (RA > 0.1%) showing cooperative and competitive associations. Green represents a competitive association and red represents a cooperative association.

Fungal co-occurrence networks among the most abundant fungal species (RA > 0.1%) are shown in Figures 6, 7. A total of 82 interaction pairs related to 25 species were obtained from the T0 timepoint, *P. kluyveri* (four linkages), *I. terricola* (four linkages), *B. caroliniana* (eight linkages), *Filobasidiales* sp. (eight linkages), *A. pullulans* (four linkages), *C. xylopsoci* (four linkages), and *A. japonicus* (four linkages) exhibited higher degrees of linkage with other species (Figure 6). The species *P. kluyveri* cooperated with *A. pullulans* and competed with *I. terricola*. *Aureobasidium pullulans* competed with *A. japonicus* and *I. terricola*. *Botrytis caroliniana* competed with *C. xylopsoci* and *Filobasidiales* sp. *Candida xylopsoci* cooperated with *Filobasidiales* sp. and *Cryptococcus victoriae*.

As shown in Figure 7, a total of 89 interaction pairs related to 26 species were obtained from the T1 timepoint; *P. kluyveri* (10 linkages), *I. terricola* (10 linkages), *B. caroliniana* (seven linkages), *Filobasidiales* sp. (10 linkages), *A. pullulans* (10 linkages), *C. xylopsoci* (seven linkages), and *A. japonicus* (10 linkages) exhibited higher degrees of linkage with other species. At the T1 timepoint, the species *Filobasidiales* sp. was observed to cooperate with *Pichia kluyveri* and *Candida xylopsoci*, whereas *Filobasidiales* sp. was observed to compete with *I. terricola*. *Aspergillus japonicus* cooperated with *I. terricola* and *Cryptococcus* sp., whereas *A. japonicus* competed with *P. kluyveri* and *A. pullulans*. *Issatchenkia terricola* competed with *Filobasidiales* sp. and *A. pullulans*.

### Correlation Analysis Between the Fungal Diversity on the Grape Berry Surface and Quality Parameters of Grape Berries

**Figure 8:**
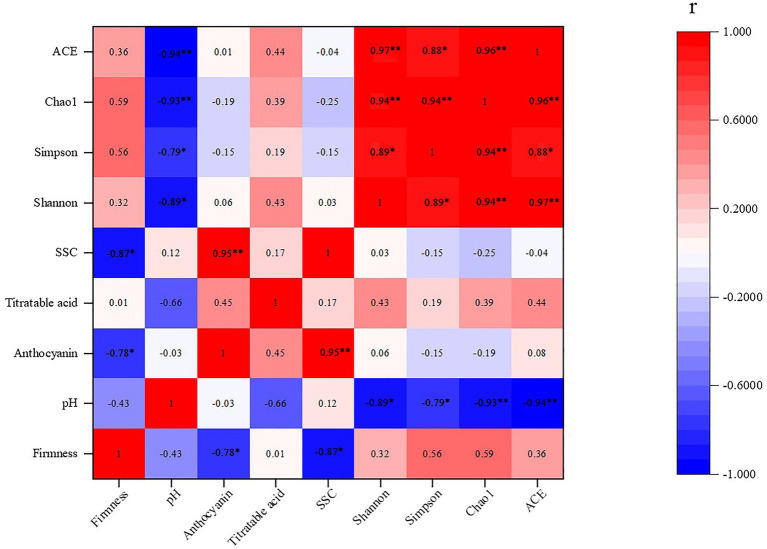
Correlation between the fungal diversity on the grape berry surface and quality parameters of the grape berries. ^∗, ∗∗^ indicate significant correlations at *p* < 0.05 and 0.01, respectively.

The Pearson correlation analysis was used to verify the correlations between the experimental indicators, such as firmness, pH, SSC, titratable acid, anthocyanin, and the Shannon, Simpson, Chao1, and ACE indexes, and the indicators of correlation are listed in Figure 8. The firmness of the grape flesh showed a positive correlation with the Chao1, Simpson, ACE, and Shannon indexes, as well as with titratable acid. The firmness of the grape flesh showed a significant negative correlation with anthocyanin and SSC. Moreover, the pH of the grape juice showed a significant negative correlation with the ACE, Chao1, Simpson, and Shannon indexes. Likewise, anthocyanin of grapes had a significant positive correlation with SSC. Additionally, titratable acid showed a positive correlation with the ACE, Chao1, Simpson, and Shannon indexes, and a negative correlation was observed between pH and titratable acid.

## Discussion

Organic farming is important to maintain or even improve soil quality (Hondebrink et al., 2017). Although organic farming is more eco-friendly and can improve the quality of grapes, it cannot meet the requirements for high grape yields in a short period. In recent years, to maintain high yields and protect the environment, the replacement of chemical fertilizers with organic ones has received more and more attention (Geng et al., 2019). In this study, we showed that the SSC of grape berries treated with organic fertilizer was higher than that of control grapes, and grape berries produced higher TAC with organic fertilizer (Figure 2). Furthermore, increased application of organic fertilizer along with reduced chemical fertilizer for 2 years induced the most significant improve the contents in the SSC and TAC of grape compared to the control grapes from T0 and T1 timepoints.

Fertilizer practices can have an impact on microbial diversity. Agricultural practices affect the community structure of phyllosphere fungal communities associated with Carménère grapevines. Grapes under organic agricultural management had more fungal species than grapes under conventional agricultural management (Castaneda et al., 2018). There are significant differences in the microbial community compositions on the surface of conventional and organic grapes (Leff and Fierer, 2013). Moreover, significant differences in fungal populations were observed in conventional and organic apples. Several unique taxa have been exclusively detected in organic apples (Abdelfattah et al., 2016a). We observed different level of fungal diversity in the fungal taxonomies obtained from the three fertilizer treatments at two timepoints (Figure 3). Also, we found that the Simpson and ACE indexes of the three fertilizer treatments at the T0 timepoint were significantly higher than those of the three fertilizer treatments at the T1 timepoint (Table 2). These results indicated that samples of the three fertilizer treatments at T0 timepoint shared a more diverse of fungal community than samples of the three fertilizer treatments at the T1 timepoint. These results indicate that room temperature storage reduces fungal diversity on the grape berry surface. Furthermore, the Simpson and ACE indexes of A.O and B.O samples (increased organic fertilizer and reduced chemical fertilizer for 1 and 2 years, respectively) were both higher than the CH samples (application of common chemical fertilizer for 2 years). These results indicate that increasing the application of organic fertilizer and reducing the application of chemical fertilizer in the grape rhizosphere can increase the fungal diversity on the grape berry surface. The majority of vine-associated microbial assemblages originate in the soil, and their distribution reflects the influence of vineyard management (Zarraonaindia et al., 2015). Morrison-Whittle et al. (2017) found 24.63% of the fungal genera which were common to soil and on the grape surface. This result was maybe due to the increased organic matter content in the soil that occurred through the application of organic fertilizer, thereby improving the soil properties and increasing the soil fungal diversity. Aboveground (leaves, flowers, and grapes) fungal communities shared a greater proportion of taxa with soil communities (Zarraonaindia et al., 2015), so the fungal diversity on the grape berry surface increased.

Ascomycota was the most highly detected phylum in this study (94.41%). The high percentage of *Ascomycota* coincided with results from other studies of *Kyoho* grape berries (80.24%; Abdelfattah et al., 2016b) and wine grape (77.00%; Carmichael et al., 2017). Gao et al. (2019) found that the main genera of four different wine grape cultivars were *Aureobasidium*, *Botrytis*, *Alternaria*, *Cladosporium*, *Mucor*, *Hanseniaspora*, and *Cryptococcus*, but their abundances in the different cultivars changed significantly. *Sclerotinia* sp., *Cladosporium* sp., *A. pullulans*, and *A. alternate* were the dominant fungal species in study of Kecskemeti et al. (2016). In our study, the predominant genera of the samples from the T0 timepoint were *Botrytis*, *Cladosporium*, *Cryptooccus*, *Pichia*, and *Aspergillus*. Correspondingly, the predominant genera of the samples from the T1 timepoint were *Pichia*, *Issatchenkia*, *Botryis*, *Cladosporium*, and *Cryptococcus*. The dominant genera of the three fertilizer treatments were almost unchanged between the T0 and T1 timepoints, but their abundances changed significantly. The components of the core fungal communities were significantly different between samples from the T0 and T1 timepoints (Figures 5D,E), indicating that the epiphytic fungal community on the grape berry surface at the T0 timepoint was significantly different from that of T1 timepoint.

Different fungal communities on the fruit surface can affect the fruit safety. Peri-urban apples samples carried potential pathogenic risks, whereas rural apple maintained more diverse fungal communities on their surfaces (Shen et al., 2018). We analyzed the dominant genera and species of the three fertilizer treatments from the T0 and T1 timepoints with RAs greater than 0.05%. We found that the RAs of *Aureobasidium* and *Candida* in the B.O samples were significantly higher than that of the CH samples at both timepoints. The RAs of *Aureobasidium* and *Candida* in the A.O samples were also higher than that of the CH samples at both timepoints, but the difference was not significant (Table 3). The epiphytic fungi living in plant tissues, particularly *Aureobasidium* (Ferreira-Pinto et al., 2006), *Candida* (Mewa-Ngongang et al., 2019), and *Pichia* (Mewa-Ngongang et al., 2019), play essential roles in disease control. *Aureobasidium pullulans* was used as a biocontrol agent against *B. cinerea* on grapes (Wang et al., 2018a). Certain fungal genera enriched in the B.O and A.O samples have been reported to exhibit protective functions. Therefore, the soil environment by organic fertilizer may provide certain advantages in maintaining healthier fungal communities on grape surfaces. *Botrytis*, *Cladosporium*, *Cryptococcus*, *Aspergillus*, and *Penicillium* were enriched in the CH samples. Significantly, several of these genera have been reported to exhibit pathogenic features and may be related with fruit decay. Specifically, *Penicillium* and *Aspergillus* are wildly reported to cause fruit deterioration and mycotoxin contamination. *Botrytis* causes gray mold on grapevines and strawberries (Haidar et al., 2016; Dowling et al., 2017). *Penicillium* causes grape losses (Platania et al., 2012; Parafati et al., 2015). *Cladosporium* was most common and important fungal agent of grape bunch rot (Mahdian and Zafari, 2016). *Aspergillus japonicus* is an important black *Aspergillus* species that may cause grape black rot (Xanthopoulou et al., 2018; Apud et al., 2019). So we suggested that CH samples carried more potential microbiological risks. These results indicated that the increased application of organic fertilizer and reduced application of chemical fertilizer to grapes could increase the RAs of beneficial fungal species on the grape surface. Increased organic fertilizer and reduced chemical fertilizer for 2 years had the most significant effect on increasing the beneficial fungal species on the grape surface.

Inter-species interactions are extremely important in shaping fungal dynamics (Shen et al., 2018). The network analysis results are shown in Figures 6, 7 demonstrate potential intra-species relationships. We observed that *A. pullulans* was competitive with *I. terricola* and *A. japonicas*. *Aureobasidium pullulans* was cooperative with *P. kluyveri* and *C. xylopsoci*. *Candida xylopsoci* competed with *B. caroliniana*. The fungal species with biological control activity cooperated against the pathogenic fungal species (Shen et al., 2018). pH significantly affected the fungal community structure, which was similar to the results of soil fungal and archaeal community structures (Wang et al., 2019) and soil bacterial community structures (Wang et al., 2018b) in an ongoing antimony mine area. The pH of the grape juice was significantly positively correlated with the Shannon, Simpson, Chao1, and ACE indexes (Figure 8). The Shannon, Simpson, Chao1, and ACE indexes were positively correlated with firmness and titratable acidity, but the correlations were not significant. Consequently, the pH of the grape juice can be regarded as a key factor affecting the fungal richness and diversity on the grape berry surface. In our past study, we found that increasing the application of organic fertilizer can significantly affect the pH of the grape juice (Zhao et al., 2020). Ren et al. (2019) also found that the fungal community structure of Pinggu peach trees was significantly correlated with pH.

In summary, increasing the application of organic fertilizer to the root zone of grapes decreased the pH of the grape juice and increased the soluble solids of the grapes, and went on to increase the fungal diversity and RAs of *P. kluyver*, *A. pullulans*, and *C. xylopsoci* (beneficial fungi) on the surface of the grape berries at T0 and T1 timepoints. The positive effect of increased organic fertilizer and reduced chemical fertilizer for 2 years was the most significant. Moreover, significant differences were revealed between the two assessment times (T0 and T1). After 8 days of storage at room temperature, the fungal diversity on the surface of the grape berries significantly decreased. The correlation analysis suggested that the pH of the grape juice was significantly negatively correlated with fungal diversity parameters.

## Data Availability Statement

The datasets generated can be found in the NCBI SRA under the Bioproject: ID PRJNA678874.

## Author Contributions

LW, ZL, and KY designed the study. LW performed the experiments and was involved with writing the manuscript. JF, HL, and KY contributed to revising the manuscript. FZ and BZ participated in lab work and helped with data analysis. All authors contributed to the article and approved the submitted version.

### Conflict of Interest

The authors declare that the research was conducted in the absence of any commercial or financial relationships that could be construed as a potential conflict of interest.
