# Supplementary material for: Increased Organic Fertilizer and Reduced Chemical Fertilizer Increased Fungal Diversity and the Abundance of Beneficial Fungi on the Grape Berry Surface in Arid Areas
Source: Front Microbiol. 2021 May 7;12:628503. doi: 10.3389/fmicb.2021.628503 (PMC8139630; doi:10.3389/fmicb.2021.628503)
Supplement: Supplementary file 1 [file Table_1.DOCX]

**Supplementary Figure 1**. Daily air temperature and precipitation from April 2017 to October 2017 and from April 2018 to October 2018
